# Factors Affecting the Implementation of Electronic Antiretroviral Therapy Adherence Monitoring and Associated Interventions for Routine HIV Care in Uganda: Qualitative Study

**DOI:** 10.2196/18038

**Published:** 2020-09-10

**Authors:** Jessica E Haberer, Lindsey Garrison, John Bosco Tumuhairwe, Robert Baijuka, Edna Tindimwebwa, James Tinkamanyire, Bridget F Burns, Stephen Asiimwe

**Affiliations:** 1 Center for Global Health Massachusetts General Hospital Boston, MA United States; 2 Department of Medicine Harvard Medical School Boston, MA United States; 3 Kabwohe Clinical Research Centre Kabwohe Uganda; 4 Department of Urban Studies and Planning Massachusetts Institute of Technology Boston, MA United States

**Keywords:** adherence, antiretroviral therapy, electronic monitoring, implementation science

## Abstract

**Background:**

High, sustained adherence to HIV antiretroviral therapy (ART) is critical for achieving viral suppression, which in turn leads to important individual health benefits and reduced secondary viral transmission. Electronic adherence monitors record a date-and-time stamp with each opening as a proxy for pill-taking behavior. These monitors can be combined with interventions (eg, data-informed adherence counseling, SMS-based adherence support, and/or alarms) and have been shown to improve adherence in multiple settings. Their use, however, has largely been limited to the research context.

**Objective:**

The goal of the research was to use the Consolidated Framework for Implementation Research (CFIR) to understand factors relevant for implementing a low-cost electronic adherence monitor and associated interventions for routine HIV clinical care in Uganda.

**Methods:**

We conducted in-depth qualitative interviews with health care administrators, clinicians, and ART clients about likes and dislikes of the features and functions of electronic adherence monitors and associated interventions, their potential to influence HIV care, suggestions on how to measure their value, and recommendations for their use in routine care. We used an inductive, content analysis approach to understand participant perspectives, identifying aspects of CFIR most relevant to technology implementation in this setting.

**Results:**

We interviewed 34 health care administrators/clinicians and 15 ART clients. Participants largely saw the monitors and associated interventions as favorable and beneficial for supporting adherence and improving clinical outcomes through efficient, differentiated care. Relevant outside factors included structural determinants of health, international norms around supporting adherence, and limited funding that necessitates careful assessment of costs and benefits. Within the clinic, the adherence data were felt likely to improve the quality of counseling and thereby morale, as well as increase the efficiency of care delivery. Existing infrastructure and care expenditures and the need for proper training were other noted considerations. At the individual level, the desire for good health and a welcomed pressure to adhere favored uptake of the monitors, although some participants were concerned with clients not using the monitors as planned and the influence of poverty, stigma, and need for privacy. Finally, participants felt that decisions around the implementation process would have to come from the Ministry of Health and other funders and would be influenced by sustainability of the technology and the target population for its use. Coordination across the health care system would be important for implementation.

**Conclusions:**

Low-cost electronic adherence monitoring combined with data-informed counseling, SMS-based support, and/or alarms have potential for use in routine HIV care in Uganda. Key metrics of successful implementation will include their impact on efficiency of care delivery and clinical outcomes with careful attention paid to factors such as stigma and cost. Further theory-driven implementation science efforts will be needed to move promising technology from research into clinical care.

**Trial Registration:**

ClinicalTrials.gov NCT03825952; https://clinicaltrials.gov/ct2/show/NCT03825952

## Introduction

High and sustained adherence to antiretroviral therapy (ART) is critical for achieving HIV viral suppression, which in turn leads to important individual health benefits and reduced secondary viral transmission [1,2]. However, the attention paid to adherence in clinical settings varies. Health care providers may ask about missed doses or perform pill counts, but these measures tend to overestimate adherence [3-5]. Pharmacy refill data is less biased and can improve detection of nonadherence [6]; yet all of these adherence measures are obtained with clinic visits and potentially after viremia has led to drug resistance [7]. Electronic adherence monitoring involves smart pill containers that record a date-and-time stamp with each opening as a proxy for pill-taking behavior. Real-time versions of electronic adherence monitors contain modems that transmit these data via cellular networks for internet-based review. Although limited by the need to use the monitor for each dose, these monitors provide daily adherence records that can trigger timely adherence interventions, potentially before the loss of viral suppression.

Several studies have suggested the effectiveness of electronic monitoring for adherence support. One study conducted in China found an increase in ART adherence when short message service (SMS) reminders triggered by real-time detection of missed doses were combined with data-informed counseling (ie, adherence records were used at clinic visits to develop solutions to adherence challenges) [8]. A similar study of triggered SMS reminders in South Africa (that did not include supported counseling) observed a decrease in sustained treatment interruptions [9]. Further, a randomized controlled trial in Uganda tested real-time adherence monitoring plus SMS reminders to patients and SMS notifications to social supporters (ie, friends or family who could help support adherence) and found improved average adherence and a reduction in sustained interruptions [10]. Other studies have used non–real-time electronic adherence monitoring data to inform counseling and also found increased ART adherence [11,12]. Importantly, these devices have been shown to be feasible and acceptable in these and other settings, although sometimes with technical challenges [13].

Despite this evidence of improved adherence and promise for HIV outcomes, electronic adherence monitors and associated interventions have largely remained in the research context—a fate common among mobile health (mHealth) interventions [14]. One clear initial barrier to implementation has been cost. Electronic monitors have traditionally cost more than US $100 each and require data transmission and hosting fees; SMS may necessitate additional development and other fees. Recently, a low-cost electronic monitor with integrated SMS messaging was developed with total costs of less than US $30 per patient per year. To contextualize these costs, a modeling analysis found that adherence monitoring–based interventions could be considered cost-effective in sub-Saharan Africa at up to $50 per person-year [15]. Intervention adoption, however, is influenced by many factors other than cost and can be holistically considered through the Consolidated Framework for Implementation Research (CFIR) [16]. The CFIR includes 5 domains: (1) intervention characteristics (eg, design, cost), (2) outer setting (eg, organizational knowledge of patient needs, external policies), (3) inner setting (eg, culture, relative prioritization), (4) individual characteristics (eg, beliefs about the intervention, self-efficacy), and (5) process (eg, planning for implementation, engaging leaders).

Here we present an exploratory analysis guided by CFIR and involving ART clients, clinicians, and health care administrators in which we sought to understand factors relevant for implementing electronic adherence monitoring and associated interventions for routine HIV clinical care in Uganda.

## Methods

### Study Setting

This study was based at the Kabwohe Clinical Research Centre (KCRC) in rural southwestern Uganda. The KCRC ART Clinic provides PEPFAR-subsidized care for more than 6000 individuals living with HIV. It is a health center level IV facility, which also provides other comprehensive primary health care services and is governed by the Ministry of Health (MoH) and a district health officer. Specialized care is available through regional and national referral hospitals, and community-level care is provided through lower-level health facilities and community health workers. The research team met with a local community advisory board and KCRC leadership prior to initiating the study and incorporated their feedback in the study design.

### Study Participants

We stratified ART clients by duration of ART use (less than vs more than 6 months) and residence type (rural vs periurban); within these categories, we identified clients randomly (ie, every 10th patient attending clinic) to understand the average experience in the clinic. Given the hierarchical nature of the Ugandan health care system, we identified up to 5 health care administrators/clinicians from each of the following cadres: MoH officials; regional referral hospital administrators; district health officers; and health center III/IV clinic administrators, physicians, nurses, and ART adherence counselors. Inclusion criteria for all participants were aged over 18 years and engagement in HIV care through one of the above-defined roles. Additionally, ART clients had to have HIV infection per clinic records and own a cellular phone (familiarity with cellular technology was felt important to inform their input on the intervention). Exclusion criteria for all participants were unwillingness or inability to provide informed consent.

### Electronic Adherence Monitor and Associated Interventions

**Figure 1:**
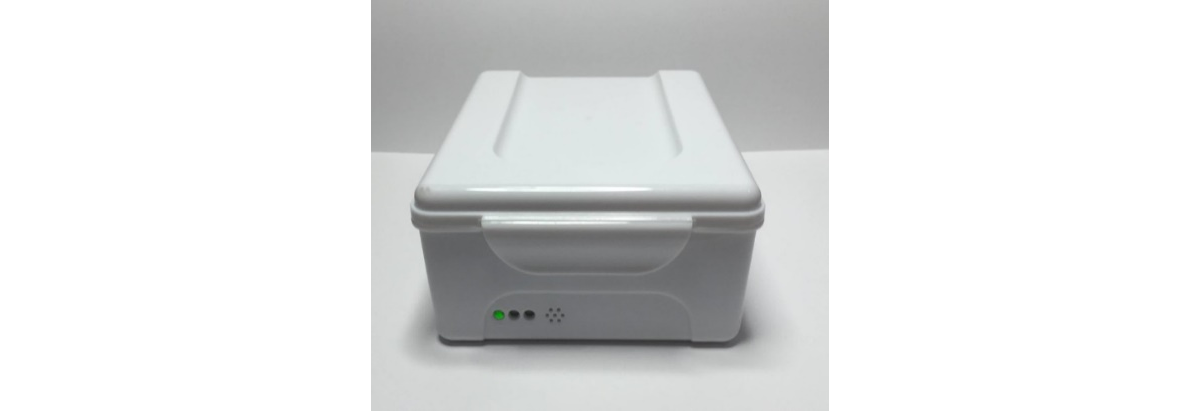
The evriMED real-time adherence monitor.

We studied the evriMED electronic adherence monitor (Wisepill Technologies, Figure 1), which can function with or without real-time data transmission. It can be paired with any combination of the interventions presented in Table 1.

**Table 1:** Interventions to be combined with electronic adherence monitoring.

Intervention	Description
Data-informed counseling	Records of monitor openings are reviewed on a smartphone, tablet, or computer and discussed at each clinic visit to identify specific challenges and develop effective solutions to overcome future adherence barriers.
One-way scheduled SMS^a^ to patients	SMS messages are sent to patients daily to encourage adherence (eg, through establishing the habit of daily pill taking and/or reminding patients that the clinic supports them). SMS are sent regardless of the recorded adherence.
One-way triggered SMS	When real-time monitors are used, SMS are sent to patients when one or more doses are taken late or missed. The SMS are sent to the patient and/or a social supporter (ie, a person who knows the patient’s HIV status and is willing to provide support).
Two-way SMS	Both scheduled and triggered SMS allow for a callback from study staff to provide support directly at that time.
Alarms	Monitors are programmed to make audio-visual alerts when it is time to take medication.

^a^SMS: short message service.

### Qualitative Interviews

**Figure 2:**
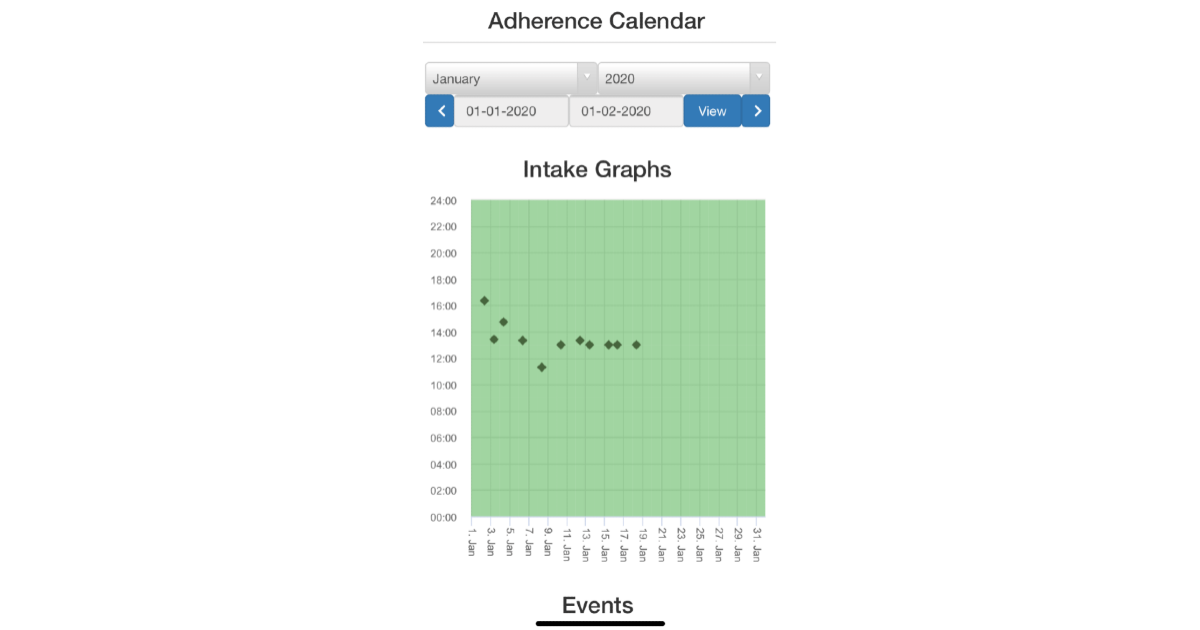
Adherence data display: each dot indicates the date and time of a monitor opening as a proxy of medication ingestion.

Interviews were conducted by authors JBT and RB, who are both bilingual in the local language (Runyankole) and English and highly experienced, well-trained male qualitative research assistants. One-time interviews were digitally recorded for later transcription and took place in private settings; most occurred in the study office or near the clinic, although all MoH interviews were conducted in the participants’ offices in Kampala, and some participants were interviewed at home, per their preference. Interviews with health care administrators/clinicians were conducted in English, which is commonly used in professional settings; interviews with ART clients were conducted in Runyankole or English per participant preference. Interviews began with an introduction to the research assistants, followed by statements of no conflicts of interest, a desire for honest perceptions (favorable or unfavorable), and the overall goals of the study. Participants were then asked for basic demographic data. A description of the electronic adherence monitors, associated interventions, evidence for their use, logistical requirements, and costs was subsequently read to participants (Multimedia Appendix 1). Participants were also shown an electronic adherence monitor and the software interface for displaying adherence data (Figure 2). Interview guides (Multimedia Appendix 2 and 3) were designed to obtain unbiased impressions of the technology and its potential for supporting ART in routine care, while also assessing each of the 5 domains in the CFIR. The guides were tailored for anticipated perspectives of health care administrators/clinicians versus ART clients. Initial questions in both guides asked about likes and dislikes of the features and functions of monitor and associated interventions and were followed by questions about their potential to influence HIV care, suggestions on how to measure their value, and recommendations for their use in routine care. Health care administrators/clinicians were also asked about the technology in relation to other health care priorities (ie, the outer setting). Questions were informally pretested with KCRC staff and clients and revised to ensure clarity and utility.

Research assistants wrote debriefs after each interview to capture body language, participant mood, and any other nonverbal aspects of the interviews. Transcripts were reviewed for quality among authors LG, BFB, JBT, and RB and corrected as needed. Participants were interviewed until thematic saturation was achieved.

### Analysis

We used an inductive, content analysis approach [17] to explore factors that could influence the implementation of electronic adherence monitors plus associated interventions. We identified the aspects of CFIR [16] that participants indicated were most relevant to their context and potential implementation of the technology. In an iterative process, authors LG and JEH read the first 20% of transcripts, formulated codes, and assembled and pilot-tested a codebook. LG subsequently used the codebook to code the qualitative data, which was entered into qualitative analysis software (Dedoose, SocioCultural Research Consultants LLC). JEH and LG then developed categories by characterizing core concepts, developing labels, writing operational definitions, and selecting illustrative quotes from the interviews. Themes were reviewed with the qualitative research assistants but not participants to ensure accurate reflection of the participants’ stated perspectives.

### Ethics

All participants provided written informed consent. This study was reviewed and approved by the institutional review boards at the Mbarara University of Science and Technology, Ugandan National Council for Science and Technology, and Partners Healthcare. The study was registered with ClinicalTrials.gov [NCT03825952].

## Results

### Participant Characteristics

We interviewed 34 health care administrators/clinicians with a mean age of 37 (SD 10) years; 56% (19/34) were female. Four administrators worked in the MoH, while 5 worked in regional referral hospitals and 5 at the district level; 5 clinicians each were doctors, nurses, and adherence counselors. We also interviewed 15 ART clients with a mean age of 40 (SD 13) years; 60% (9/15) were female, and 67% (10/15) had taken ART for less than 6 months. A total of 60% (9/15) lived in periurban settings, and 40% (6/15) lived in rural settings. None of the individuals approached for participation declined. Interviews lasted an average of 51 minutes.

### Overview

Table 2 presents the main factors participants felt would influence implementation of the adherence monitors and associated interventions. The factors are organized within the 5 domains of CFIR. Thematic saturation was achieved with the 49 study participants. Note that all perspectives reflect hypothetical use of the monitors and associated interventions.

**Table 2:** Factors influencing implementation of adherence monitors and associated interventions according to the Consolidated Framework for Implementation Research domains. The category of participant endorsing each factor is indicated per the footnote.

Intervention	Outer	Inner	Individual	Process
Metrics of value Improved monitoring and support for adherence^a^ Tool for management of viremia^b^ Improved clinical outcomes^a^ Facilitation of differentiated care^b^ Device and SMS^c^ features^a^ Real-time monitoring versus stored records^a^	Structural determinants of health^b^ International norms^b^ Funding in the context of costs and benefits^b^	Data-informed counseling^a^ Quality of counseling^a^ Clinic morale^a^ Existing infrastructure and care expenditures^b^ Facilitation of efficient care delivery^b^ Need for in-service training, staffing, and support^b^	Desire for health^a^ Desire to please clinicians^d^ Not using monitors as planned^a^ Poverty, stigma, and privacy^a^	Decision making by the Ministry of Health and other funders^a^ Sustainability^b^ Target population^a^ Coordination across the health care system^b^

^a^Both health care administrators/clinicians and antiretroviral therapy client.

^b^Health care administrators/clinicians only.

^c^SMS: short message service.

^d^Antiretroviral therapy clients only.

### Intervention

Both health care administrators/clinicians and ART clients uniformly stated that the value of the adherence monitor and associated interventions would be seen in their ability to improve knowledge of and support for adherence. They were seen as better than the current, limited approach to adherence monitoring.

The evriMED device is a good monitor in this era of technology and it will improve on monitoring of patient’s adherence because we have been relying on backward methods like pill counting, which have their own disadvantages.

District health officer, male, 54 years (health care administrator; HCA-1-023)

If I used to miss taking my drugs for days or take beyond the time I am supposed to be swallowing the pills, and after using the monitor, I find that I no longer miss or take my drugs late, I would know that the monitor has really helped me to improve my adherence.

ART client, female, 35 years (individual taking ART; ITA-1-007)

Participants stated they would support using the monitor and associated interventions if they improved clinical outcomes (eg, viral suppression). Health care administrators/clinicians felt the data could also be used to differentiate care by targeting adherence counseling only to those in need, as well as helping to determine if viremia is due to nonadherence or drug resistance. This information would enable rapid and appropriately tailored care.

I think this would make them [ART clients] to remember taking their drugs the next day before it becomes a habit and worsens the situation.

ART client, female, 45 years (ITA-1-002)

I know if people are monitored well on adherence and if people at all do adhere because of this monitor, then you are improving their livelihood. Their chances of developing resistance will be very low because they are taking their ARVs or in case they are failing, then it is very easy for you to make a decision [on how to provide care].

Physician, male, 30 years (HCA-1-030)

If people are adhering to their treatment with support of such monitor and they are taking their medicine well, we expect they should suppress...and they can be moved to the differentiated delivery model which doesn’t require them to come a lot to the clinic because now they move to the state of being stable and they decongest the clinic and they give us much time to look at the people who are very sick.

Physician, male, 42 years (HCA-1-033)

Both categories of participants identified several features of the monitors and associated interventions as advantageous for uptake, including size and secure storage capacity.

It is portable, it is clean, and it keeps all the ART client’s drugs there.

Physician, female, 39 years (HCA-1-018)

The size should remain as it is so that I put in drugs for one month... The size should not be bigger than it is because it will become bulky and difficult to carry.

ART client, female, 48 years (ITA-1-012)

What I have liked about it is that it’s like as if you go to the shop and buy a suitcase for keeping your clothes...so that they don’t get damaged, it’s the same thing I have liked about the evriMED because it will keep my drugs safely rather than keeping them in a piece of paper.

ART client, female, 45 years (ITA-1-009)

On the contrary, other participants felt the device was too big or too small, and several logistical requirements were seen as potentially limiting uptake, including the need to charge batteries. Other features could lead to unintended and undesirable HIV status disclosure (eg, flashing lights or alarms).

My concerns are about electricity. For example, power may be off and the battery of the evriMED gets low. So, what will happen? Because it will not monitor when the battery is off.

ART client, female, 55 years (HCA-1-014)

If this device makes an alarm and you tell the patient to go with drugs to a place which is crowded, say it is a party, and it is going to make an alarm, I think that will be a challenge.

ART client, male, 42 years (HCA-1-033)

Views differed on the importance of real-time data transmission versus stored records that could be reviewed during clinic visits. Opinions were guided by prioritization of optimal adherence compared with cost and staffing resources. SMS reminders were seen as a way to extend the reach and support from the clinic. Both categories of participants felt that real-time intervention would reduce the necessary focus on adherence in clinic.

Real-time is much better as far as responses are concerned because there is a patina of poor adherence which people adopt, and it keeps on recurring and when we don’t do real-time, we might lose time as well. Real-time is better, and I know the cost will come down.

Physician, male, 42 years (HCA-1-033)

We don’t have the money and if this device is coming in to increase the cost of care per patient, I don’t think it will be scalable but if it is not increasing cost per client then it will be good. Therefore, storing information and discussing it after is better.

MoH administrator, male, 42 years (HCA-1-025)

If a client receives a message informing him or her that he/she swallowed medicine very well last week; therefore, he or she should continue swallowing medicine very well. The client will feel happy that health workers are caring about him or her which will encourage client to continue swallowing his or her medicine very well.

Adherence counselor, female, 31 years (HCA-1-007)

It will influence my care in a great way because I may have forgotten to take my drugs or I am about to forget, but the SMS could remind me to take me drugs when it comes.

ART client, female, 35 years (ITA-1-012)

While participant views varied on specific features of the monitors and associated interventions, nearly all saw considerable potential for positive impact on HIV care.

### Outer Setting

Beyond the intervention, health care administrators/clinicians noted that several structural factors would influence the success or failure of the interventions. For example, unstable personal life circumstances and community-level influences, like stigma and discrimination, may not be addressed by the proposed interventions.

It is very good for settings in the village whereby routine for day work is regular, but in town where people keep on moving from one place to another it might be challenging... The assumption may not hold that people are forgetting to take their drugs. There might be other reasons that are causing them not to take their drugs including the peer influence, pill burden fatigue, denial, stigma and discrimination.

MoH administrator, male, 43 years (HCA-1-022)

That said, this category of participants recognized the developing international norms for supporting adherence. They cited the Joint United Nations Programme on HIV and AIDS (UNAIDS) 90-90-90 policy that calls for 90% viral suppression; high adherence is critical for achieving this goal and would thus favor uptake of the technology.

Now adherence is one of the issues that we are looking up in the next strategic plan to increase efficiencies... We are almost enrolling 90% of our targets, but that is not good enough to end HIV. We need to increase efficiencies in the programs, so devices like the evriMED that increase efficiencies are very necessary to achieve the end to HIV.

MoH administrator, male, 43 years (HCA-1-022)

Another major factor was funding. Participants weighed benefits versus the costs in the context of other HIV expenditures.

I think we have to discuss it and look at the budget and we have a way out of it. But when you look at the benefits and you weigh against the cost, I think it is beneficial.

Regional referral hospital administrator, female, 32 years (HCA-1-034)

If, for example, our clinic was performing at 80% in good care, it can move our performance from 80% to 90%... But still we are performing without it. I hope you understand me. I am not saying that it doesn’t have value and it can improve us... It will reduce the expenses on patients we move from first line to second line, so it has a lot of value. But I don’t know the value when you mixed it with the cost.

Physician, female, 45 years (HCA-1-029)

Overall, the health care administrators/clinicians indicated that competing demands for limited funding would necessitate documented benefit from the intervention at a low total cost.

### Inner Setting

Within the clinic, both categories of participants indicated that adherence monitor data would likely have considerable effects on counseling that would influence implementation. Most felt that the above-noted objectivity would change the dynamic between counselors and clients, allowing for more honesty and impact. The anticipated higher quality adherence counseling and more open relationship were seen as valuable for both clients and clinicians. This dynamic could improve job satisfaction for clinicians, thus motivating continued employment and continuity of care. Some participants, however, cautioned about the potentially negative impact of documented nonadherence.

When [the counselor] knows that I am adhering to my drugs well, it will also give him/her morale and encouragement to continue doing his/her job. We disturb them a lot... We don’t tell them that we got some challenges that made us adhere poorly. You remain in the village and they bring you back to the clinic when your health has already deteriorated. You start blaming the clinic, saying they have not done enough for your health or cared about you, yet it’s because of your ignorance.

ART client, female, 35 years (ITA-1-007)

Interviewer: What makes you think that it is not good for counselor seeing this information [adherence graph] and presenting it to you during your clinic visit? Participant: Because if you don’t swallow your medicine very well, then counselor will think that he/she wasted time teaching you how to swallow your medicine.

ART client, female, 35 years (ITA-1-006)

When our clients are well or when they are healthy, I also feel okay because I know that I have done something great.

Clinician, male, 58 years (HCA-1-008)

Health care administrators/clinicians stated that the effects on counseling would have to be assessed within the context of clinic infrastructure and expenditures. Because the clinic already uses SMS with clients, SMS-based interventions would be affordable and easy to implement. The price of the monitor was seen as low when considered against other routine costs (eg, transportation). However, the overall cost of the monitors plus supporting infrastructure was a concern.

The cost of messages is not on the very high side. Currently as a clinic, we already have a platform that sends messages to patients who have raised viral load, and this doesn’t cost us a lot.

MoH administrator, male, 42 years (HCA-1-028)

It is a very useful device... So, what can make me to invest is the cost vis-à-vis the number of monitors I am going to buy. And by the way the cost of the monitor together with the cost of the running infrastructure and the internet and all other things to ensure that everything is running well.

Physician, female, 45 years (HCA-1-029)

Health care administrators/clinicians emphasized that the above-noted ability to differentiate care would facilitate efficient care delivery in the clinic. For example, ART counselors would be better able to tailor their adherence counseling and allow the clients who are doing well to minimize their time in clinic. These efficiencies would help justify the cost of the monitors and SMS.

Those clients who will be found to be adhering well based on the monitor data and have no adherence issues may need no counseling while those that are doing badly in respect to adherence will be given adequate counseling or even switched to more experienced and skilled counselors for proper counseling or management.

District health office, male, 54 years (HCA-1-023)

The research will find out whether it [monitor] increases on the time each client spends with the doctor on clinic visit and it is good for the patient to spend more time with the doctor instead of rushing through.

MoH administrator, male, 42 years (HCA-1-025)

To enable uptake of the monitoring and associated interventions, participants also highlighted the need to provide in-service training. Opinions differed as to whether additional staff and/or infrastructure would be needed.

There is need to teach the clinic staff about the technology, how to use it, and how to do counseling based on that data from the monitor. If they teach staff on how to use it, there will be no challenges with clinical implementation.

Adherence counselor, female, 49 years (HCA-1-011)

The additional support the clinic might need is training of the personnel that will be involved in the use of this device/technology and providing logistics to the clinics necessary to use this technology which could be either from government or development partners. Things like tablets, computers if necessary and other accompanying equipment.

District health officer, male, 42 years (HCA-1-028)

Within the clinic setting, participants stated that the adherence monitors and associated interventions could have a significant impact on clinical care delivery, which they felt should be considered in any implementation plans.

### Individual

Both categories of participants also identified several individual-level characteristics that could impact implementation. For instance, ART clients who prioritized improved health expressed enthusiasm to use the intervention. They described a welcomed pressure to adhere.

What I have liked is that it may report you to the clinic that you are not taking your drugs well, and as a patient, this will force you to take your drugs well and live longer.

ART client, male, 37 years (ITA-1-005)

Additionally, clients indicated that the monitors would enable them to demonstrate good adherence to the health care workers. This motivation reflected their appreciation of the care they receive in clinic.

I will know that the counselor is trying to help me on the basis of that data, to make sure that I live a healthy life and I cannot feel bad about it because I will know that she/he cares about my life... This counseling will help me to change my behavior because I will know that they will keep posted with my adherence.

ART client, female, 48 years (ITA-1-012)

That said, both categories of participants felt inaccuracies may arise in the data if clients do not use the monitors as planned, which would limit the value of the adherence data.

He/she might remove the pills, take them somewhere, and forgets or some time passes before he/she swallows the medicine. I would feel there would be a camera or a way of being sure that the client has taken the drugs after opening and removing them.

Clinic administrator, female, 34 years (HCA-1-020)

Poverty was seen as a principal influencing factor. Many participants felt clients could not pay for the monitors or associated SMS or pay for electricity to charge the monitors or their cell phones to receive or send SMS. Participants were also concerned that both traditional illiteracy and technical illiteracy (ie, ability to use technology)—two indirect effects of poverty—would limit some clients’ ability to use the SMS.

[SMS] reminders in Uganda have not really yield the positive, because we have had reminders to mothers to attend antenatal... but the issue is that the phone must be charged, and the person must be with the phone and able to read the message.

MoH administrator, male, 43 years (HCA-1-022)

You know majority of patients are illiterate... so you have to continue emphasizing education on how to use the monitor. If you don’t do that then they forget in a very short time.

District health officer, male, 42 years (HCA-1-035)

Nearly all participants commented on the potential for stigma in the event of lost privacy (eg, others seeing the monitors or SMS); however, views differed on the importance of these factors in using the intervention. Opinions seemed to stem from the extent to which clients accepted their HIV diagnosis and disclosed their status to others.

So you have to educate them on the advantages and functions of these monitors. If they feel out place carrying them, they may feel stigmatized. They may feel segregated and end up not using them.

Regional referral hospital administrator, female, 32 years (HCA-1-034)

I have no problem with the evriMED bringing stigma. I don’t fear that because I am not the first person to have HIV or to be taking ART drugs. I have no problem with it.

ART client, female, 23 years (ITA-1-002)

Some people may not feel comfortable getting into their privacy in that someone knowing that there is a monitor that is recording him and information accessed by the third party, that alone may either increase stigma.

Physician, female, 23 years (ITA-1-002)

Participants felt that these individual-level factors were critical for uptake of the monitors and associated interventions. Challenges would be important to address through counseling and education.

### Process

Consistent with the above-noted emphasis on cost-effectiveness, health care administrators/clinicians described funders as playing a key role in the implementation process. Support and resources were expected from the MoH and/or other organizations contracted for HIV services delivery in Uganda (eg, USAID-supported Elizabeth Glazer Pediatric AIDS Foundation). Most ART clients and clinicians reported having insufficient funding themselves to implement the monitors or interventions.

The clinic has no funding specifically to buy this [evriMED] because it receives medicines from the Ministry of Health through national medical stores and the staff belongs to the Ministry of Health and currently some of them are employed by RHITES Southwest, which is implemented by EGPAF... one needs to market it at the ministry level.

MoH administrator, male, 42 years (HCA-1-028)

I am not willing to pay for reminder messages or anything that evriMED does.

ART client, male, 58 years (ITA-1-008)

They will think that health workers have a hidden agenda in collecting that money which may spoil image of the health workers and the facility since our facility is a government facility and they think everything is for free.

Adherence counselor, female, 31 years (HCA-1-010)

In addition to upfront costs, health care administrators/clinicians highlighted the importance of program sustainability. They indicated the need to consider long-term costs, which depend partly on monitor durability.

That is one [question], affordability, sustainability and how do we service it supposing the funder left several of these and the funders pulled out, whoever the funder may be. Can the clinic program manage to service it, sustain it, so that it can be able to continue to work?

Physician, female, 45 years (HCA-1-029)

Health care administrators/clinicians also indicated that sustainability of the monitors and associated interventions would depend on the target population. Opinions on the target population, however, varied widely in both categories of participants. Some suggested including all ART clients, while others wanted to select those with high risk or documented adherence challenges. Participants felt that large numbers of clients would limit implementation, although excluding clients could also cause challenges.

For something to be routine, it should be able to be affordable for everybody and some of our clinics are very big like 12,000 [clients]...for example, is it something which is affordable for routine care when we are even failing to have Septrin to give to patients?

Physician, female, 45 years (HCA-1-029)

But this monitor can be a backup for a specific group of people, maybe the adolescents whose adherence is difficult, those patients who are nonsuppressed, maybe the mothers with high viral load...not everybody, because I think it maybe not sustainable, because it is very expensive.

Physician, female, 45 years (HCA-1-029)

All patients on ART should use the evriMED if possible.

ART client, male, 37 years (ITA-1-005)

It’s a problem that you may not explain to every patient what the monitor is about. You may talk to those to whom you intend to give it, but patients talk to one another and they share a lot. So some may feel they are left out and feel that they are being segregated and denied a service.

Regional referral hospital administrator, female, 32 years (HCA-1-034)

If the decision to implement were made, health care administrators/clinicians advised coordination among multiple levels of health care organization through to the client to ensure successful care delivery.

There will be a need for like orientation for a capacity-building plan right from national level through the regions, districts, and the facilities. So, that has to be planned very well because people would want to know how to use the monitor and give the correct information to people.

MoH administrator, female, 41 years (HCA-1-024)

Attitude of the patients is a very important factor. You can do anything but if the patients’ attitude towards the device is bad, you waste a lot of money.

Physician, male, 30 years (HCA-1-030)

Participants took a holistic view of the implementation process, including all of the above-noted factors related to the intervention, individual, inner setting, and outer setting.

## Discussion

### Principal Findings

This qualitative study of ART clients, health care administrators, and clinicians explored factors that may influence the implementation of low-cost electronic ART monitors and associated interventions for routine HIV care in Uganda. To our knowledge, this study is the first to use an implementation science framework to explore the means to move a technology-based adherence intervention from research into clinical care. The intervention was largely seen as favorable and beneficial for improved clinical outcomes with efficient, differentiated care. Concerns centered primarily around potential for stigma, device misuse, possible need for additional resources, and cost in the setting of competing demands for limited resources.

Participants identified improvements for the intervention that could address some of their concerns. For instance, stigma could be reduced by simple alterations, such as variable monitor sizes and making alarms optional. Counseling specifically around disclosure could also alleviate concerns for stigma, and education could support fidelity of use, even with low levels of literacy among clients. Within the clinic, leveraged use of existing infrastructure and staff would increase efficiency and reduce concerns about cost. And, perhaps most importantly, demonstrated value in clinical outcomes for the lowest possible cost could position implementation well against competing demands for limited resources in HIV care. These insights are particularly valuable, as most research on electronic adherence monitoring has focused only on measurement, even when considering the context of routine clinical care [18].

The potential value of an improved approach to adherence monitoring and support was endorsed by all participants. Steady progress is being made toward the UNAIDS 90-90-90 goals, and many are already achieving the high adherence necessary for viral suppression. Yet sustained adherence remains a challenge over time. Up to one-third of clients in sub-Saharan Africa are viremic at 2 years of therapy [19] and similar numbers have stopped ART and been lost from care at 5 years [20]. Increased adherence monitoring and support could play a critical role in reaching the 10-10-10 currently eluding the current care models.

Several tuberculosis treatment programs globally have begun to implement electronic adherence monitoring and support as part of routine care [21], although little evidence has been published on the implementation of these approaches. Studies driven by implementation science frameworks could facilitate uptake, assess for fidelity of implementation, and understand the impact on clinical outcomes. These data will be critical to determine how well potential benefits translate into real-world settings. Indeed, some preliminary reports with other digital adherence monitoring approaches suggest challenges with patient engagement and accuracy [22] that will need to be systematically addressed.

### Limitations

This study has limitations. First, although we interviewed health care workers and administrators from all levels of the health care system, the ART clients came from a single site. That said, KCRC and the client population characteristics are largely reflective of ART delivery in rural East Africa. Second, study findings reflect perceived views that were not influenced by actual use of the monitors or associated interventions. Future work will involve deployment of the technology with subsequent reflections on the implementation process. Strengths of the paper include use of a comprehensive implementation science framework and in-depth exploration of factors relevant for the implementation process.

### Conclusions

In conclusion, we found that low-cost electronic adherence monitoring combined with data-informed counseling, SMS-based support, and/or alarms has potential for use in routine HIV care in Uganda. Key metrics of successful implementation will include their impact on efficiency of care delivery and clinical outcomes with careful attention paid to factors such as stigma and cost. Given that most interventions fail to progress from research to practice, further theory-driven implementation science efforts will be needed to realize the benefits of this promising technology.

## Appendix

Multimedia Appendix 1 Description of the electronic adherence monitor and associated interventions.

Multimedia Appendix 2 Qualitative interview guide for clients taking antiretroviral therapy.

Multimedia Appendix 3 Qualitative interview guide for healthcare administrators and clinicians.

## Abbreviations

ART: antiretroviral therapy
CFIR: Consolidated Framework for Implementation Research
KCRC: Kabwohe Clinical Research Centre
mHealth: mobile health
MoH: Ministry of Health
PEPFAR: President's Emergency Plan for AIDS Relief
SMS: short message service
UNAIDS: Joint United Nations Programme on HIV and AIDS
