# Supplementary material for: Factors Affecting the Implementation of Electronic Antiretroviral Therapy Adherence Monitoring and Associated Interventions for Routine HIV Care in Uganda: Qualitative Study
Source: J Med Internet Res. 2020 Sep 10;22(9):e18038. doi: 10.2196/18038 (PMC7516683; doi:10.2196/18038)
Supplement: Multimedia Appendix 1 [file jmir_v22i9e18038_app1.docx]

Multimedia Appendix 1. Description of the electronic adherence monitor and associated interventions (read to participants):

*Electronic adherence monitors*: These devices are “smart” medication containers that record when they are opened as a marker of medication use (also known as medication adherence). Records of all container openings are stored in the monitor. These records can be accessed by physically connecting the monitor to a computer, tablet, or smart phone (standard monitors) or through cellular networks (real-time monitors). The monitors can be used solely for understanding when medication may have been taken, or they can be paired with interventions. <<A research assistant then demonstrated the device and mobile app>>

*Data-informed counseling*: The data from both the standard and real-time monitors can be reviewed as a printout at each clinic visit. These data gives a day-to-day record of adherence. Gaps in adherence can be discussed in detail so that clinicians or counselors can help patients identify specific challenges and develop effective solutions to overcome barriers to adherence in the future. When presented positively, both the monitoring and the data-informed counseling can be seen as support from the clinic and it can be effective in achieving and maintaining medication adherence.

*Interventions*: In addition to the data-informed counseling, other interventions can be paired with the monitors.

- - One-way scheduled SMS to patients: SMS can be sent to patients daily or weekly to encourage adherence (for example, through establishing the habit of daily pill taking and/or reminding patients that the clinic supports them). These SMS are sent regardless of the recorded adherence.
  - One-way triggered SMS: When using real-time monitors, SMS can be sent to patients when one or more doses are taken late or missed. The SMS can be sent to the patient and/or a designated person who knows the patient’s HIV status and is willing to provide support.
  - Two-way SMS: Both scheduled and triggered SMS can allow for a call back from study staff to provide support directly at that time.
  - Alarms: The monitors can be programmed to make audio and/or visual alerts when it is time to take medication.
