# Supplementary material for: Factors Affecting the Implementation of Electronic Antiretroviral Therapy Adherence Monitoring and Associated Interventions for Routine HIV Care in Uganda: Qualitative Study
Source: J Med Internet Res. 2020 Sep 10;22(9):e18038. doi: 10.2196/18038 (PMC7516683; doi:10.2196/18038)
Supplement: Multimedia Appendix 2 [file jmir_v22i9e18038_app2.docx]

Multimedia Appendix 2. Qualitative interview guide for clients taking ART.

1. Please tell me what you think of the evriMED monitor. Tell me a little bit about what it does (this probe is meant to get baseline understanding of what the participant understands about the device). Specifically, what do you like? What do you not like?

*Probe on recommendations for device improvement (e.g., size, alarms).*

1. In what ways do you think the monitor could be helpful for you in getting HIV care? I am referring to your usual visits to the clinic, not anything having to do with a study.

*Probe on individual benefits for pill taking, experiences during clinic visits. How might this change over time (e.g., with long-term use)?*

1. What are your concerns about how the evriMED monitor could affect your care? Again, I am referring to your usual visits to the clinic, not anything having to do with a study.

*Probe on privacy, stigma, expense (willingness to pay for the monitor, ability to pay for SMS to respond to the clinic), time spent in clinic*

1. Data from the monitor would look like this <show mock-up>. What do you think about a counselor seeing this information and presenting it to you during your clinic visit?

*Probe on counseling messages, interactions with the counselor*

1. What do you think about using the data from the monitor to send supportive SMS (for example, if you missed a dose or simply once a week regardless of how you take your pills)? To whom should the SMS go? When should they be sent? What should they say? Should HIV or ART be mentioned? Would you like to send SMS back to the clinic?

*Probe on privacy, stigma, expense*

1. As I mentioned, when the monitor records openings, it can store them for your clinician to see when you go there next. Or, it can send them in real-time, but real-time communication adds more cost to the monitor and for the SMS. Which do you think is better? Why?
2. This intervention depends on technology, and all technology has the potential to fail at times. For example, the cellular network may not be working well and records may come late, leading clinicians to misunderstand your true adherence or sending unnecessary SMS. Please tell me how that possibility affects your opinion of the intervention.
3. Tell me how much you think the monitor could influence your HIV care. How much do you think counseling based on the monitor data could influence your care? How much do you think the SMS could influence your care?

*Probe on this value in relation to the current standard of care*

1. What recommendations do you have for using the evriMED monitor in usual HIV clinic visits (not research)? Who should use it? How long should they use it?

*Probe on recommendations compared to other clinic improvements the participant may want to see*

1. How would you know if the monitor was helping you or other patients?

*Probe on acceptability, improved confidence in care, better adherence data and/or lab values (CD4, viral load)*

1. Is there anything else you would like to tell me about the monitor or any interventions that could be associated with it? Are there other ways we could use this technology to support ART?
2. Are there other improvements you would like to see in the way the HIV clinic is run?

*Remind participants that we are asking for their opinions, but cannot necessarily influence the clinic itself.*

**READ:** Thank you for your participation. The interview is now over.
