# Supplementary material for: Factors Affecting the Implementation of Electronic Antiretroviral Therapy Adherence Monitoring and Associated Interventions for Routine HIV Care in Uganda: Qualitative Study
Source: J Med Internet Res. 2020 Sep 10;22(9):e18038. doi: 10.2196/18038 (PMC7516683; doi:10.2196/18038)
Supplement: Multimedia Appendix 3 [file jmir_v22i9e18038_app3.docx]

Multimedia Appendix 3. Qualitative interview guide for healthcare administrators and clinicians.

1. Please tell me what you think of the evriMED monitor. Tell me about what it does (this probe is meant to get baseline understanding of what the participant understands about the device). Specifically, what do you like? What do you not like?

*Probe on recommendations for device improvement (e.g., size, alarms)*

1. In what ways do you think the monitor could be helpful for routine HIV clinical care?

*Probe on individual benefits for patients, benefits for clinic flow and outcomes*

1. What are your concerns about the evriMED monitor for routine clinical care? Again, I am referring to your usual visits to the clinic, not anything having to do with a study.

*Probe on privacy, stigma, expense (for the clinic and/or patients, including cost of the monitor and SMS), staffing/logistics*

1. Data from the monitor would look like this <show mock-up>. What do you think about using the data from the monitor to influence adherence counseling?

*Probe on impact on clinic flow, counseling messages*

1. What do you think about using the data from the monitor to send supportive SMS? To whom should the SMS go? When should they be sent? What should they say? Should HIV or ART be mentioned? Should they be one-way or two-way?

*Probe on privacy, stigma, expense, staffing/logistics*

*Do you think that there could be challenges with clinical implementation? What sort of additional support might the clinic need to utilize this monitor in routine clinical care?*

1. As I mentioned, when the monitor records openings, it can store them for clinicians to see when patients come in next. Or, it can send them in real-time, but real-time communication adds more cost to the monitor and for the SMS. Which do you think is better? Why?
2. This intervention depends on technology, and all technology has the potential to fail at times. For example, the cellular network may not be working well and records may come late, leading clinicians to misunderstand a patient’s true adherence or sending unnecessary SMS. Please tell me how that possibility affects your opinion of the intervention.
3. Tell me how much value you think the monitor has for influencing HIV care and treatment. How much do you think counseling based on the monitor data could influence care? How much do you think the SMS could influence care?

*Probe on this value in relation to other clinical programs or interventions (in the clinic or elsewhere in the healthcare system, real or theoretical)*

1. What recommendations do you have for using the evriMED monitor in routine HIV clinical care (not research)? Who should use it? How long should they use it? What factors would influence your decision to invest or not invest in the monitor? How can you (in your current role) influence the decision to make this technology available to patients?

*Probe on priorities for program implementation*

1. How would you want to measure the value of the monitor?

*Probe on use of the monitor (e.g., number of monitors used), patient and/or provider acceptability, adherence data, clinical outcomes (e.g., number of people staying on first-line ART)*

*What type and how many patients would you like to use the monitor?*

*Why would you want them to use the monitor?*

1. Is there anything else you would like to tell me about the monitor or any interventions that could be associated with it?
2. Are there other ways in which you would prefer to see ART adherence managed in the clinic?

*Remind participants that we are asking for their opinions, but cannot necessarily influence the clinic itself.*

**READ:** Thank you for your participation. The interview is now over.
